# Gait Parameters and Postoperative Pain Following Total Ankle Arthroplasty Using Wearable Motion Sensors: A Cross-Sectional Study

**DOI:** 10.7759/cureus.78392

**Published:** 2025-02-02

**Authors:** Ryo Yoshikawa, Noriyuki Kanzaki, Ryoma Matsuno, Wataru Saho, Ryoga Kashima, Risa Harada, Ryosuke Kuroda, Yoshitada Sakai

**Affiliations:** 1 Division of Rehabilitation Medicine, Kobe University Graduate School of Medicine, Kobe, JPN; 2 Department of Orthopedic Surgery, Kobe University Graduate School of Medicine, Kobe, JPN; 3 Department of Rehabilitation Medicine, Kobe University Hospital, Kobe, JPN; 4 Department of Physical Medicine and Rehabilitation, Kobe University Hospital, Kobe, JPN

**Keywords:** gait analysis, postoperative pain, total ankle arthroplasty, wearable device, wearable technology

## Abstract

Objectives

Total ankle arthroplasty (TAA) is increasingly performed to alleviate pain and improve function in patients with end-stage ankle osteoarthritis. This study aimed to evaluate the gait characteristics of patients with TAA using a motion sensor-based system and to investigate the relationship between postoperative pain and gait parameters.

Methods

This cross-sectional study included patients at least three months post-TAA. The evaluated parameters included sex, age, postoperative period, and the dorsiflexion/plantarflexion range of motion (ROM) of the ankle. Gait analysis was performed using a six-axis inertial sensor attached to both shoes to measure stride speed, heel-strike angle, toe-off angle (TOA), pronation angle, foot progression angle, vertical height, swing width, and stride length normalized to height. Pain was assessed using the Self-Administered Foot Evaluation Questionnaire (SAFE-Q). Comparisons were made between the surgical and contralateral sides, and the correlations between pain scores and measured angles were examined.

Results

Thirty-eight patients (mean age: 76 ± 5 years) were evaluated. The surgical side demonstrated significantly lower TOA (48.9° vs. 59.0°) and plantarflexion ROM (35.5° vs. 49.6°) compared with the contralateral side (p < 0.001). Although the pain scores showed no correlation with ROM, TOA on the surgical side showed a positive correlation with pain scores (r = 0.379, p < 0.05).

Conclusions

The surgical side exhibited reduced plantarflexion ROM and smaller TOA compared with the contralateral side. Additionally, greater postoperative pain was associated with lower TOA on the surgical side. Therefore, motion sensor-based gait analysis can be a valuable tool for tailoring rehabilitation strategies and improving outcomes following TAA.

## Introduction

Ankle osteoarthritis (OA) is a degenerative condition characterized by cartilage degradation in the ankle joint, leading to joint space narrowing, swelling, and pain. It affects approximately 6% of the population [1]. Joint deformity can limit mobility, making walking difficult and reducing the activities of daily living (ADLs) and quality of life (QOL). Total ankle arthroplasty (TAA) is a surgical procedure that involves removing the damaged surfaces of the ankle joint and replacing them with an artificial joint. TAA can effectively alleviate ankle joint pain and restore ankle joint function [2-4]; it has become a standard surgical treatment for patients with end-stage ankle OA [5,6]. However, residual postoperative pain can adversely affect patients’ ADL and QOL [7]. Moreover, the optimal gait pattern following TAA remains unclear, and the relationship between postoperative pain and gait characteristics needs to be elucidated further.

In recent years, a motion sensor-based gait analysis system has been developed to measure various gait parameters. This system evaluates position and angle-level gait parameters by analyzing the acceleration and angular velocity data of shoes during walking and running. It can be easily measured by simply attaching a small device to the shoe and has demonstrated excellent relative validity for measuring gait parameters [8].

In this study, we aimed to investigate the gait patterns of patients following TAA using a motion sensor-based gait analysis system and further examine the impact of postoperative pain on gait.

## Materials and methods

Ethics

This study was conducted in accordance with the principles of the Declaration of Helsinki for Human Research and was approved by the Ethics Committee of Kobe University Graduate School of Medicine (B230083). Informed consent was obtained from the patients or their legal guardians.

Study design and patients

This cross-sectional study included patients who had undergone TAA for OA. Gait analysis was performed during postoperative follow-up visits to our outpatient clinic between August 2023 and October 2024. Patients who required revision surgery, had undergone bilateral ankle surgeries, were within three months postoperatively, or had a history of rheumatoid arthritis, psychiatric disorders, or dementia were excluded.

Assessment of gait function using six-axis inertial sensors

**Figure 1:**
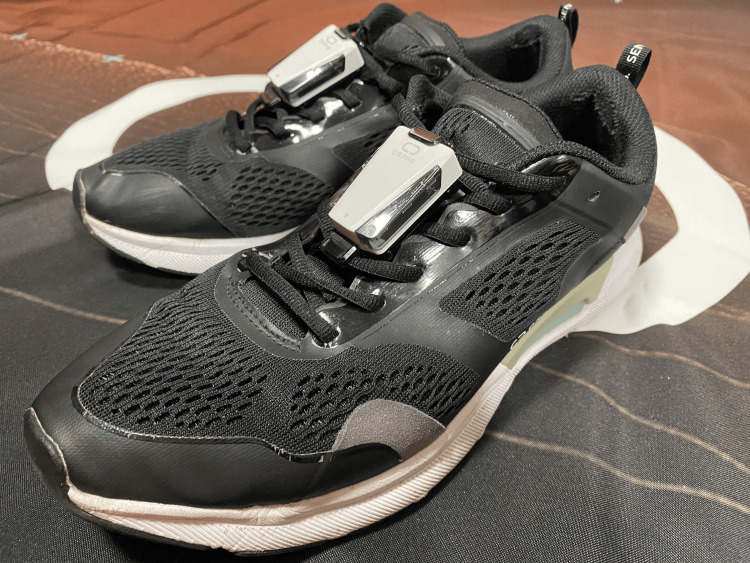
Attachment of six-axis inertial sensors Six-axis inertial sensors (white devices) were attached to the insteps of both shoes.

Gait function was evaluated using a six-axis inertial sensor (ORPHE CORE MEDICAL; ORPHE Inc., Tokyo, Japan), which recorded acceleration and angular velocity at a sampling frequency of 200 Hz. Each sensor weighed approximately 20 g and measured 45 × 29 × 14 mm. The sensors were capable of detecting triaxial acceleration (X, Y, Z) and triaxial angular velocity (X, Y, Z). The coordinate system for the sensors was defined as follows: for the right foot, the X-axis was oriented laterally toward the right hand, the Y-axis pointed anteriorly, and the Z-axis was directed vertically upward. For the left foot, the coordinate system was mirrored along the YZ plane relative to the right foot. Sensors were attached to the instep of both shoes, ensuring that the Y-axis of the sensor aligned parallel to the longitudinal axis of the foot in the horizontal plane (Figure 1). The patients were instructed to walk a distance of 10 m at a normal speed, during which gait data were collected.

Evaluated factor

Patient demographic data, including sex, age, height, body weight, BMI, and postoperative duration, were collected. The ankle dorsiflexion angle (DFA) and plantarflexion angle (PFA) were measured by physical therapists. The acceleration and angular velocity data recorded using six-axis inertial sensors were processed using a proprietary gait analysis software (ORPHE ANALYTICS MEDICAL; ORPHE Inc.) to calculate gait parameters. These gait parameters included stride speed and bilateral measures such as heel-strike angle (HSA), toe-off angle (TOA), pronation angle, foot progression angle, vertical height (VH), swing width (SW), and stride length normalized to height (SL/H) (Table 1). Speed, relative position, and relative angle were calculated using an inertial navigation system based on zero-velocity updates, a method that corrects drift errors by assuming zero velocity during the stance phase [9]. These parameters were derived by integrating the angular velocity and acceleration data obtained from the sensors.

**Table 1:** Gait parameters calculated using a gait analysis software FPA, foot progression angle; HSA, heel-strike angle; PA, pronation angle; SL/H, stride length normalized to height; SS, stride speed; SW, swing width; TOA, toe-off angle; VH, vertical height

Evaluation parameters	Unit	Explanation
SS	m/s	The average walking speed in a single measurement, calculated based on the movement speed per step
HSA	degree	The angle between the sole of the foot and the ground surface at landing. A higher value indicates a greater ability to land with the toes lifted.
TOA	degree	The angle between the sole of the foot and the ground surface during foot push-off. A higher value indicates the ability to push off with the heel lifted.
			PA	degree	The angle at which the foot rolls inward from landing to full contact with the ground surface. A larger value indicates a greater inward roll.
FPA	degree	The angle between the long axis of the foot and the direction of movement. A positive value indicates that the toes are pointing outward.			
			VH	cm	The height at which the foot is elevated during the swing phase.
SW	cm	The maximum distance the foot moves outward during the swing phase
SL/H		The distance between the heel contact of one foot and the next heel contact of the same foot, adjusted by dividing by height

The measurements obtained from both sides were compared between the surgical side and the contralateral side. Pain scores were evaluated using the pain subscale of the Self-Administered Foot Evaluation Questionnaire (SAFE-Q), a patient-reported outcome measure [10,11]. The SAFE-Q score indicates that a higher score means lower pain.

Statistical analysis

Statistical analysis was performed using Prism 10 for macOS (GraphPad Software, San Diego, CA, USA). The Wilcoxon signed-rank test was used to compare the measurements between the surgical side and the contralateral side. Spearman’s rank correlation coefficient was used to examine the correlation between the measured angles and pain scores. Statistical significance was set at a p-value of <0.05.

## Results

A total of 38 participants (six men and 32 women) were included in the study, with a mean age of 75.9 ± 4.6 years. The mean BMI was 24.5 ± 3.5 kg/m², while the mean walking speed was 1.1 ± 0.2 m/s.

Comparison of measured angles

**Figure 2:**
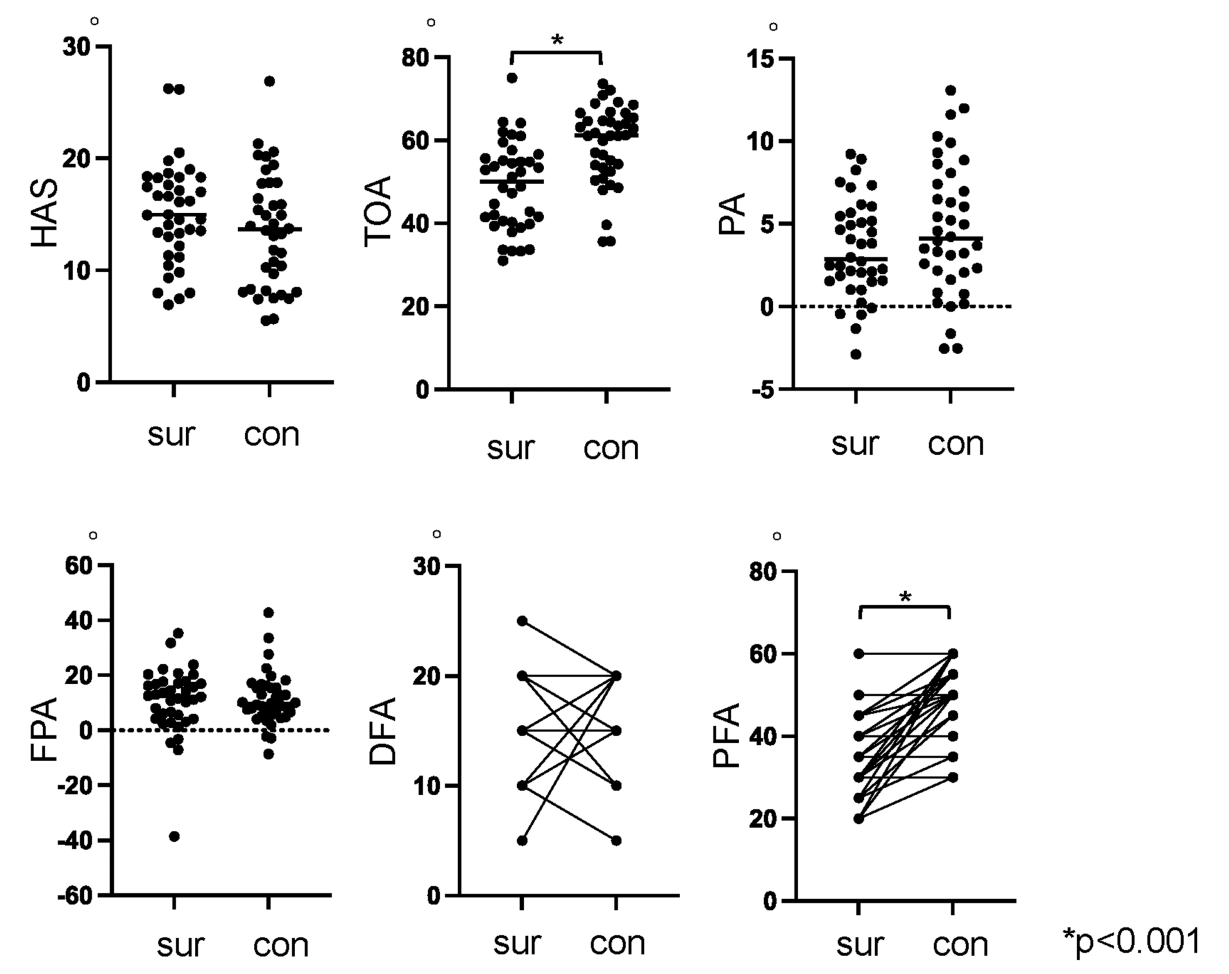
Comparison of gait angles between the surgical and contralateral sides The measured angles on the surgical side and contralateral side are presented as dot plots, with horizontal lines indicating the median value for each group. For DFA and PFA, the corresponding values within the same patient are connected by lines. con, contralateral; DFA, dorsiflexion angle; FPA, foot progression angle; HSA, heel-strike angle; PA, pronation angle; PFA, plantarflexion angle; sur, surgical; TOA, toe-off angle

The comparison of measured angles between the surgical side and contralateral side (Figure 2) showed that the TOA was significantly smaller on the surgical side (48.9° ± 10.6°) compared with the contralateral side (59.0° ± 9.5°; p < 0.001). Similarly, the PFA was significantly smaller on the surgical side (35.5° ± 9.0°) compared with the contralateral side (49.6° ± 7.7°; p < 0.001). No significant differences were observed for other measured angles between the two sides.

Comparison of measured lengths

**Figure 3:**
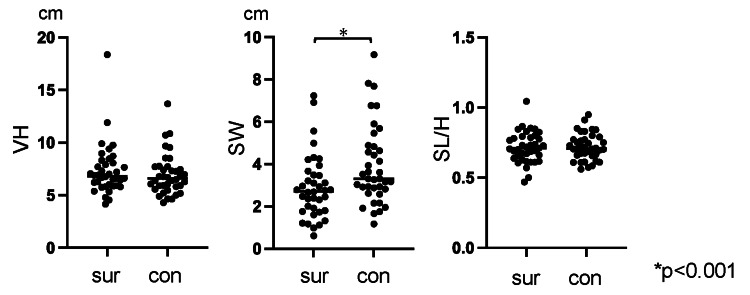
Comparison of measured lengths between the surgical and contralateral sides The measured lengths in the surgical side and contralateral side are presented as dot plots, with horizontal lines indicating the median value for each group. con, contralateral; SL/H, stride length normalized to height; sur, surgical; SW, swing width; VH, vertical height

With regard to the measured lengths (Figure 3), the SW was significantly smaller on the surgical side (2.9 ± 1.5 cm) compared with the contralateral side (3.9 ± 1.9 cm; p < 0.001). No significant differences were observed between the two sides in terms of VH or SL/H.

Correlation with pain scores

Next, the correlations between the pain scores on the SAFE-Q and various parameters were analyzed. Table 2 shows the correlations between pain scores and patient demographics or walking speed. No significant correlations were observed in any of these variables.

**Table 2:** Correlation between pain scores and patient demographics/walking speed PD, postoperative duration; SS, stride speed

Variable	Spearman correlation coefficient	95% CI	p-value
PD	0.277	−0.057 to 0.555	0.092
Age	−0.107	−0.420 to 0.230	0.524
Height	0.024	−0.307 to 0.349	0.889
Body weight	−0.069	−0.389 to 0.265	0.68
BMI	−0.163	−0.466 to 0.175	0.33
SS	0.273	−0.061 to 0.552	0.098

Table 3 presents the correlations between pain scores and measured angles. A significant positive correlation was found only for the TOA on the surgical side (r = 0.379, p < 0.05) and the HAS on the contralateral side (r = 0.349, p < 0.05). No significant correlations were observed for other angles, including DFA and PFA.

**Table 3:** Correlation between pain scores and measured angles DFA, dorsiflexion angle; FPA, foot progression angle; HSA, heel-strike angle; PA, pronation angle; PFA, plantarflexion angle; TOA, toe-off angle

Parameter	Surgical side			Contralateral side		
	Spearman correlation coefficient	95% CI	p-value	Spearman correlation coefficient	95% CI	p-value
HSA	0.062	−0.272 to 0.383	0.71	0.349	0.023 to 0.608	0.032
TOA	0.379	0.057 to 0.629	0.019	0.066	−0.268 to 0.386	0.694
PA	0.136	−0.202 to 0.444	0.417	0.041	−0.291 to 0.365	0.805
FPA	0.117	−0.220 to 0.429	0.483	0.02	−0.310 to 0.346	0.905
DFA	0.112	−0.225 to 0.425	0.503	0.039	−0.293 to 0.363	0.816
PFA	0.143	−0.195 to 0.450	0.393	−0.156	−0.461 to 0.182	0.35

Table 4 displays the correlations between pain scores and measured lengths. Significant positive correlations were observed only for the SL/H on the surgical side (r = 0.464, p < 0.01) and the VH on the contralateral side (r = 0.407, p < 0.05). Raw data used for the analyses in this study are provided in Appendix A.

**Table 4:** Correlation between pain scores and measured lengths SL/H, stride length normalized to height; SW, swing width; VH, vertical height

Parameter	Surgical side			Contralateral side		
	Spearman correlation coefficient	95% CI	p-value	Spearman correlation coefficient	95% CI	p-value
VH	0.013	−0.317 to 0.340	0.938	0.407	0.091 to 0.649	0.011
SW	0.164	−0.174 to 0.467	0.325	−0.066	−0.386 to 0.268	0.693
SL/H	0.464	0.160 to 0.688	0.003	0.208	−0.129 to 0.503	0.209

## Discussion

This study is the first to evaluate gait patterns following TAA using a motion sensor-based gait analysis system attached to the instep of a shoe. The findings revealed that patients post-TAA exhibited a reduced TOA and plantarflexion range of motion (ROM) on the surgical side compared with the contralateral side, as well as a smaller SW. TAA is known to preserve ankle joint motion compared with ankle arthrodesis, providing a more physiological treatment approach [12]. However, previous studies have reported that limitations in plantarflexion ROM often persist postoperatively [13]. Consistent with these findings, our study observed no significant differences in dorsiflexion ROM between the surgical and contralateral sides, while plantarflexion ROM was significantly reduced on the surgical side. This reduction in plantarflexion ROM likely contributed to the decreased TOA observed during walking. A diminished TOA has been associated with reduced walking speed in previous studies [14,15]. Regarding SW, it is possible that patients reduced the SW on the surgical side to avoid pain. This suggests that adjusting toward the values of the contralateral side may contribute to gait improvement. These results suggest that improving the TOA on the surgical side may be an important target for rehabilitation following TAA. Addressing this issue may not only involve improving ankle motion but also enhancing knee flexion and hip extension angles during the late stance phase or utilizing insoles to optimize biomechanics. Future studies are warranted to explore effective rehabilitation strategies aimed at improving these parameters and optimizing postoperative outcomes.

Additionally, this study explored the relationship between postoperative pain experienced during the performance of ADLs and gait patterns, revealing distinct gait characteristics in patients with residual pain following TAA. These patients demonstrated a reduced TOA and shorter SL on the surgical side, whereas the contralateral side exhibited a decreased HSA and a lower VH during walking. The results on the contralateral side suggest compensatory actions to reduce pain on the surgical side. Contrary to expectations, no significant correlation was observed between pain and dorsiflexion or plantarflexion ROM. Given that pain in this study was assessed using the SAFE-Q, which reflects pain levels during the performance of ADLs [11], the findings suggest that dynamic assessments may be more effective compared with static evaluations in improving postoperative patient satisfaction. Although this study cannot definitively establish the causal relationship between pain and gait patterns, the ability to quantify gait characteristics in patients experiencing postoperative pain has significant clinical implications. Future research should conduct longitudinal evaluations within the same cohort to monitor temporal changes in gait parameters. Such investigations could aid in identifying optimal gait characteristics that contribute to pain reduction, thereby guiding the development of targeted rehabilitation strategies to improve postoperative outcomes.

Gait analysis serves as a valuable tool for objectively quantifying the extent of functional improvement following TAA. However, previous studies often relied on extensive systems such as three-dimensional gait analysis [2,16,17], which, although highly accurate, are impractical for routine clinical use. By contrast, the device used in this study offers significant advantages in terms of convenience and accessibility. Attaching the device to the shoe allows for quick and easy quantification of gait parameters, making it increasingly applicable in clinical practice [8,18]. Moving forward, utilizing this device to monitor patients’ progress from preoperative to postoperative stages holds significant potential. It could aid in identifying effective rehabilitation strategies, optimizing treatment plans, and enhancing patient motivation. This streamlined approach to gait analysis may bridge the gap between advanced biomechanical evaluations and practical, real-world applications in patient care. However, as the device alone cannot assess the movement of the knee, hip, and trunk, a comprehensive physical evaluation by physicians or physical therapists is essential.

Limitations

This study has several limitations. First, as a cross-sectional study, it limits the ability to evaluate longitudinal changes in gait and pain following TAA. Future studies should adopt a longitudinal design to explore gait parameters associated with pain improvement and to develop more effective rehabilitation strategies. Second, there is a mix of patients with and without OA in the contralateral ankle. This should be further examined by increasing the sample size and conducting a stratified analysis. Finally, this study primarily focused on the overall motion of the foot and ankle, without considering the movement of the knee or hip, or lower limb muscle strength. Future research should examine the correlation between these parameters and gait characteristics. Despite these limitations, this study utilized a device capable of conveniently measuring gait in clinical settings by attaching it to the shoe’s instep. The specific gait characteristics of patients who experienced TAA with postoperative pain were determined using this device. These findings may serve as a reference for improving postoperative rehabilitation and developing more effective treatment approaches.

## Conclusions

This study provides valuable insights into the gait patterns of TAA patients by utilizing a clinically applicable motion sensor-based system. The results highlight significant differences in the gait mechanics between the surgical and contralateral sides, with reduced plantarflexion ROM and a smaller TOA observed on the surgical side. Additionally, greater postoperative pain was associated with a lower TOA on the surgical side, suggesting that this specific gait parameter may reflect a diminished capacity for effective propulsion during walking. These findings underscore the potential of motion-sensor-based gait analysis as a powerful tool for assessing gait dysfunction in TAA patients. By identifying specific gait deficits, clinicians may be able to personalize rehabilitation strategies aimed at reducing pain and improving functional outcomes, ultimately enhancing the QOL for individuals undergoing TAA.

## Appendices

Appendix A

**Table 5:** Raw data used for analysis con, contralateral; DFA, dorsiflexion angle; FPA, foot progression angle; HSA, heel-strike angle; PA, pronation angle; PD, postoperative duration; PFA, plantarflexion angle; SL/H, stride length normalized to height; SS, stride speed; sur, surgical; SW, swing width; TOA, toe-off angle; VH, vertical height

Subject	SAFE-Q_pain	PD (months)	Sex (0: female, 1: male)	Age (years)	Height (cm)	Body weight (kg)	BMI (kg/m²)	SS (m/s)	HSA_sur (°)	HSA_con (°)	TOA_sur (°)	TOA_con (°)	PA_sur (°)	PA_con (°)	FPA_sur (°)	FPA_con (°)	DFA_sur (°)	DFA_con (°)	PFA_sur (°)	PFA_con (°)	VH_sur (cm)	VH_con (cm)	SW_sur (cm)	SW_con (cm)	SL/H_sur	SL/H_con
1	61.4	6	0	79	148	55	25.11	0.78	16.20	8.18	33.27	55.14	4.07	3.11	22.27	8.76	20	20	30	50	6.69	6.08	1.32	2.15	0.61	0.61
2	100.0	24	0	74	153	52	22.21	0.99	11.35	10.40	53.44	56.51	2.11	6.30	1.24	18.17	15	20	40	40	7.03	10.85	3.67	5.69	0.71	0.66
3	73.3	12	0	72	153	47	20.08	1.03	18.27	14.92	55.65	53.34	-2.90	2.06	-7.15	8.56	20	20	45	55	6.61	6.41	2.01	3.31	0.70	0.72
4	89.4	24	0	71	152	56	24.24	1.18	6.94	10.26	53.67	63.14	4.64	3.50	10.30	3.52	10	15	50	50	4.15	4.90	2.45	3.17	0.73	0.71
5	83.9	12	0	80	153	70	29.90	0.99	19.00	16.42	61.35	61.09	9.22	6.97	14.12	16.75	20	10	40	40	6.46	6.41	6.92	9.18	0.67	0.67
6	85.2	3	0	72	154	56	23.61	0.88	7.99	7.43	39.85	66.56	2.06	2.59	14.51	8.04	15	20	25	60	11.90	7.40	1.80	5.46	0.61	0.61
7	91.7	24	0	80	148	76	34.70	0.94	18.38	13.09	33.62	48.04	2.28	2.33	16.75	11.96	15	15	25	45	6.77	5.92	0.98	1.17	0.64	0.67
8	88.4	12	0	71	145	63	29.96	0.94	9.35	7.80	41.62	35.67	8.26	11.99	35.26	42.70	20	10	20	30	5.83	7.70	2.69	3.27	0.67	0.66
9	100.0	24	0	78	153	72	30.76	1.09	13.53	17.83	56.60	61.77	6.06	9.30	17.82	15.31	20	20	40	50	7.06	8.54	3.94	2.92	0.73	0.75
10	70.8	6	0	81	149	54	24.32	0.83	14.98	8.07	44.67	35.64	1.57	7.41	4.11	4.03	10	15	35	50	5.80	5.02	2.79	3.52	0.57	0.59
11	45.2	6	1	75	168	73	25.86	0.78	9.84	5.50	33.39	54.28	5.68	8.85	17.65	15.35	15	15	45	55	8.08	5.91	4.31	7.82	0.50	0.58
12	82.6	3	0	73	158	50	20.03	1.30	16.62	19.42	61.01	68.55	5.45	-2.55	20.72	-8.63	20	20	45	60	7.20	6.70	2.31	3.16	0.83	0.84
13	55.8	12	0	78	152	69	29.86	0.98	12.19	13.26	31.00	64.61	-1.34	4.97	6.07	12.93	15	20	40	50	6.53	6.27	3.21	5.90	0.66	0.70
14	74.1	12	0	80	148	58	26.48	1.25	14.57	15.38	54.50	61.21	1.50	0.76	4.22	6.61	10	15	20	50	5.34	5.19	1.92	2.82	0.69	0.71
15	76.1	12	1	80	168	68	24.09	0.96	13.00	7.52	40.30	52.49	6.18	3.98	31.77	27.65	20	20	40	55	6.22	5.94	1.73	3.64	0.63	0.61
16	100.0	12	1	79	165	60	22.04	1.25	14.54	15.89	59.55	57.03	8.92	13.07	16.14	12.78	15	20	40	50	7.71	7.46	3.49	4.89	0.78	0.76
17	41.2	6	0	75	152	61	26.40	0.90	7.98	8.06	42.05	39.63	1.02	10.29	12.15	33.59	15	20	30	50	8.97	4.64	1.21	4.83	0.47	0.61
18	100.0	12	0	67	157	44	17.85	1.18	14.94	7.48	64.40	61.07	4.50	-1.64	15.80	-2.87	20	20	45	50	6.02	4.66	2.36	4.14	0.87	0.72
19	75.8	12	0	80	160	58	22.66	1.28	13.31	13.74	52.89	73.63	7.20	9.91	-38.58	9.06	15	15	30	50	4.56	5.83	3.11	6.76	0.77	0.83
20	85.3	24	0	70	149	53	23.87	1.29	16.12	17.76	75.04	70.88	7.53	6.49	10.78	9.87	20	20	60	60	6.62	6.79	2.93	3.04	0.85	0.85
21	93.3	3	0	77	151	62	27.19	1.04	18.32	15.79	62.06	62.84	1.86	-2.53	2.70	6.67	15	10	30	30	5.87	13.69	4.21	4.43	0.74	0.70
22	56.3	24	0	83	154	62	26.14	0.86	7.47	8.32	52.42	50.34	2.74	2.17	6.64	17.19	10	5	50	50	4.79	5.57	3.10	1.66	0.61	0.62
23	90.6	3	0	82	145	56	26.63	0.84	13.66	14.17	33.73	59.88	-0.45	0.84	5.55	5.53	15	20	30	55	5.39	10.72	1.17	2.94	0.62	0.56
24	32.2	3	0	63	160	54	21.09	1.12	18.30	9.68	40.59	69.22	-0.09	5.43	2.38	10.01	15	20	20	60	7.33	8.51	1.76	3.61	0.69	0.67
25	93.6	6	0	69	161	66	25.46	1.13	13.38	11.80	42.81	64.71	5.08	4.23	23.87	7.58	10	20	30	60	5.91	5.22	1.12	2.16	0.73	0.69
26	65.0	12	0	74	160	54	21.09	1.40	26.23	26.88	54.85	72.10	5.17	8.07	11.54	1.95	20	20	35	35	8.33	9.54	4.25	7.69	0.85	0.91
27	79.6	3	0	81	157	52	21.10	1.08	17.46	13.56	48.58	65.44	2.97	3.23	12.90	4.84	15	20	35	55	6.89	6.83	1.60	4.64	0.72	0.71
28	100.0	24	0	75	148	46	21.00	1.08	16.67	21.30	47.29	54.01	1.54	5.22	11.18	19.74	5	20	40	50	6.27	7.28	2.75	2.58	0.69	0.70
29	47.0	3	0	81	155	64	26.64	1.14	14.06	10.77	41.50	49.24	2.45	0.22	16.94	12.81	20	15	25	35	9.90	5.48	0.61	1.91	0.66	0.73
30	72.8	24	0	78	142	52	25.79	1.34	17.02	11.57	51.15	64.02	3.79	0.01	13.78	-2.24	10	20	30	50	5.76	4.30	3.46	3.94	0.85	0.95
31	79.2	24	0	76	151	50	21.93	1.15	18.68	13.93	57.62	50.85	-0.49	6.04	-3.26	15.58	20	20	30	50	18.37	6.77	7.24	4.57	0.62	0.79
32	93.9	3	0	80	154	51	21.50	1.08	20.52	20.28	38.96	63.49	1.01	1.62	8.07	4.48	15	20	30	55	7.93	7.31	2.67	2.88	0.78	0.75
33	100.0	3	1	78	165	64	23.51	1.53	17.14	18.98	55.18	61.19	4.93	8.63	20.22	22.51	20	15	40	50	8.48	9.69	2.96	3.31	1.05	0.77
34	65.6	3	0	72	152	54	23.37	1.27	26.16	20.58	39.34	68.88	7.34	4.58	12.53	10.13	15	20	40	55	7.66	7.73	2.48	1.95	0.82	0.80
35	100.0	24	0	81	151	50	21.93	1.34	11.20	17.84	54.81	64.40	2.15	3.33	17.10	16.23	20	15	30	45	9.41	6.97	2.39	2.93	0.79	0.83
36	83.3	3	1	74	169	70	24.51	1.07	10.44	14.95	37.91	48.56	3.82	3.69	20.33	5.17	15	15	30	50	8.73	6.50	2.79	1.75	0.71	0.74
37	100.0	24	1	71	178	68	21.46	1.28	19.78	20.16	48.93	66.84	2.47	11.60	3.02	9.32	25	20	40	50	9.77	7.77	5.56	6.77	0.80	0.77
38	86.1	24	0	76	157	63	25.56	1.33	17.63	5.67	64.16	66.56	0.24	0.16	-4.56	7.40	10	15	40	50	6.91	6.08	4.99	2.62	0.78	0.84
